# Preoperative Thrombocytopenia May Predict Poor Surgical Outcome after Extended Hepatectomy

**DOI:** 10.1155/2018/1275720

**Published:** 2018-11-01

**Authors:** Mohammad Golriz, Omid Ghamarnejad, Elias Khajeh, Mohammadsadegh Sabagh, Markus Mieth, Katrin Hoffmann, Alexis Ulrich, Thilo Hackert, Karl Heinz Weiss, Peter Schirmacher, Markus W. Büchler, Arianeb Mehrabi

**Affiliations:** ^1^Department of General, Visceral, and Transplantation Surgery, University of Heidelberg, Heidelberg, Germany; ^2^Liver Cancer Center Heidelberg (LCCH), Heidelberg, Germany; ^3^Department of Gastroenterology and Hepatology, University of Heidelberg, Heidelberg, Germany; ^4^Institute of Pathology, University of Heidelberg, Heidelberg, Germany

## Abstract

**Background:**

It is a novel idea that platelet counts may be associated with postoperative outcome following liver surgery. This may help in planning an extended hepatectomy (EH), which is a surgical procedure with high morbidity and mortality.

**Aim:**

The aim of this study was to evaluate the predictive potential of platelet counts on the outcome of EH in patients without portal hypertension, splenomegaly, or cirrhosis.

**Methods:**

A series of 213 consecutive patients underwent EH (resection of ≥ five liver segments) between 2001 and 2016. The association of preoperative platelet counts with posthepatectomy liver failure (PHLF), morbidity (based on Clavien-Dindo classification), and 30-day mortality was evaluated using multivariate analysis.

**Results:**

PHLF was detected in 26.3% of patients, major complications in 26.8%, and 30-day mortality in 11.3% of patients. Multivariate analysis revealed that the preoperative platelet count is an independent predictor of PHLF (odds ratio [OR] 4.4, 95% confidence interval [CI] 1.3–15.0,* p*=0.020) and 30-day mortality (OR 4.4, 95% CI 1.1–18.8,* p*=0.043).

**Conclusions:**

Preoperative platelet count is associated with PHLF and mortality following extended liver resection. This association was independent of other related parameters. Prospective studies are needed to evaluate the predictive role and to determine the impact of preoperative correction of platelet count on postoperative outcomes after EH.

## 1. Introduction

Extended hepatectomy (EH) is the only curative treatment for large primary or bilobar metastatic hepatic malignancies that improves long-term survival [1, 2]. Surgical developments, better patient selection, and improvements in perioperative care have increased the number of EH procedures being performed [3, 4]. However, the rate of postoperative morbidity is high following EH, especially posthepatectomy liver failure (PHLF) [5, 6]. Preoperative predictive factors of PHLF may play an important role in assessing the risk of post-EH morbidity and mortality.

Several studies have evaluated different predictive factors for PHLF and other postoperative clinical outcomes [7–12]. Recently, the association of the perioperative (preoperative or immediate postoperative) platelet count with PHLF and postoperative mortality has been investigated [13–16]. However, findings have been controversial; some studies have shown a negative association between perioperative platelet counts and postoperative morbidity and mortality, while findings from other studies have indicated no association [13, 15, 17]. Although low platelet count is related to intraoperative poor outcome such as bleeding, it may have a direct impact on posthepatectomy outcomes by promoting liver regeneration and lowering the risk of PHLF. To the best of our knowledge, the association between preoperative platelet count and postoperative outcome has not been investigated exclusively in EH, which has a higher postoperative morbidity and mortality compared with minor hepatectomy.

The aim of this study was to investigate the association of the preoperative platelet count and postoperative clinical outcomes following EH in patients without portal hypertension, splenomegaly, or cirrhosis. To do this, we investigated the effect of preoperative thrombocytopenia on PHLF, morbidity, and mortality after EH.

## 2. Patients and Methods

### 2.1. Study Population

We investigated all patients who underwent liver resection to treat primary, metastatic, or benign liver disease at the department of General, Visceral, and Transplantation Surgery at the University of Heidelberg between October 2001 and September 2016. All patients were followed up until September 2017. Only patients who underwent EH were included in the study. EH was defined as resection of five or more hepatic segments based on the Brisbane 2000 classification [18]. Patients under 18 years old and patients who underwent a two-stage hepatectomy (portal vein embolization or associated liver partition and portal vein ligation for staged hepatectomy) were excluded. At the end, a total of 213 patients were included in our study. Furthermore, preoperative imaging reports, intraoperative flowmetry, and postoperative histopathological examinations were screened to assess splenomegaly, portal hypertension, and cirrhosis, respectively. Demographic and baseline clinical characteristics, as well as data on the surgical procedure and perioperative course, were prospectively collected and analyzed. This study was approved by the independent ethics committee of the University of Heidelberg. All procedures were conducted in accordance with the most recent revision of the Declaration of Helsinki.

### 2.2. Definition and Classification of Postoperative Outcomes

PHLF was diagnosed and graded (grade A, B, or C) according to the proposed definition by the International Study Group of Liver Surgery (ISGLS) [19]. Briefly, PHLF was defined as an increased international normalized ratio (INR), the need for coagulation factors to maintain normal INR, and hyperbilirubinemia on or after postoperative day 5. Hyperbilirubinemia was defined as a serum bilirubin concentration greater than 1 mg/dl and increased INR was defined as an INR greater than 1.2. In patients with preoperative hyperbilirubinemia or increased INR, PHLF was defined as an increase in serum bilirubin levels or INR on or after postoperative day 5.

The severity of postoperative morbidities were classified as grade I to V based on the Clavien-Dindo classification [20]. Grade I and II morbidities were defined as minor and grade III and IV morbidities were defined as major. Postoperative mortality was defined as all-cause death occurring within the first 30 days after surgery.

### 2.3. Preoperative Evaluations

All preoperative clinical evaluations including medical history, physical examination, and laboratory findings were recorded. All patients underwent cross-sectional contrast-enhanced computed tomography or magnetic resonance imaging of the chest, abdomen, and pelvis to assess the resectability of the tumor and to plan the hepatectomy. The preoperative platelet count was measured on the day of surgery and thrombocytopenia was defined as a platelet count <150 x 10^9^/L.

### 2.4. Statistical Analysis

Statistical analysis was performed using IBM SPSS Statistics for Windows, Version 22.0 (IBM Corp. Released 2013. Armonk, NY). Categorical data were presented as frequencies and proportions, and continuous data were presented as means ± standard deviations. Categorical data were compared using chi-square test of association or Fisher's exact test. Continuous data were compared using Student's t-test. Univariate and multivariate logistic regression analyses were performed to determine independent preoperative predictive factors of PHLF, major morbidity, and 30-day mortality. Variables with a* p *value <0.1 from the univariate analysis were included in the multivariate regression analysis. Results of univariate and multivariate analyses were reported as odds ratio (OR) with 95% confidence interval (CI). If thrombocytopenia was confirmed in the multivariate analysis, a comparison between patients with platelet counts <150 x 10^9^/L and platelet counts ≥150 x 10^9^/L was performed. One-year and three-year patient survival were analyzed using the Kaplan-Meier method. Patients who were lost to follow up were censored. The mean patient survival in the two groups was compared using the log-rank test. A two-sided* p *value less than 0.05 was considered significant in all analyses.

## 3. Results

The mean age of patients was 60.8±11.7 years and 50.7% were female. Primary hepatic malignancy was the most common indication for EH (57.7% of patients), and 35.8% of patients received preoperative systemic chemotherapy. PHLF was detected in 26.3% of patients, major complications (grade III–IV) in 26.8% of patients, and 30-day mortality in 11.3% of patients. Detailed patient demographics and clinical data are shown in Table 1.

Seventeen patients (8.0%) had a preoperative platelet count of <150 x 10^9^/L (mean platelet count=122.3±22.3 x 10^9^ per L), and the remaining 196 (92.0%) patients had a preoperative platelet count of ≥150 x 10^9^/L (mean platelet count=315.5±114.0 x 10^9^ per L). Baseline characteristics and clinical outcome of the patients with preoperative thrombocytopenia are shown in Table 2. Nine of 17 patients (52.9%) with preoperative platelet count <150 x 10^9^/L were diagnosed with primary liver malignancy (cholangiocarcinoma). Furthermore, preoperative imaging, intraoperative flowmetry, and postoperative histopathological examinations revealed no splenomegaly, portal hypertension, or cirrhosis in the thrombocytopenia group. There were only seven patients with Child-Pugh score A cirrhosis in platelet count ≥150 x 10^9^/L group. The postoperative intensive care unit (ICU) stay was longer in the preoperative platelet count <150 x 10^9^/L group (16.7 ± 9.5 days versus 8.0 ± 14.5 days,* p*=0.017). Furthermore, in the group with platelet count of <150 x 10^9^/L PHLF, major complications and 30-day mortality were detected in 58.8%, 35.3%, and 35.3% of patients, respectively (Table 2).

### 3.1. Predictive Value of Preoperative Platelet Count

To investigate the impact of the preoperative platelet count on postoperative outcomes including PHLF, morbidity, and 30-day mortality, we performed univariate and multivariate regression analysis. Univariate analysis (Table 3(a)) revealed that patients with a preoperative platelet count of <150 x 10^9^/L are significantly more at risk of PHLF (OR 4.7, 95% CI 1.7–12.9,* p*=0.003). According to univariate analysis, indication of EH, intraoperative blood loss, transfusion of red blood cells or fresh frozen plasma, and operation time also had a significant impact on PHLF. In contrast, multivariate regression only revealed preoperative thrombocytopenia as an independent preoperative predictor of PHLF (OR 4.4, 95% CI 1.3–15.0,* p*=0.020).

According to multivariate analysis, patient age (OR 1.1, 95% CI 1.0–1.1,* p*=0.001), metastatic liver disease (OR 2.4, 95% CI 1.1–5.1,* p*=0.026), and operation time (OR 1.2, 95% CI 1.0–1.5,* p*=0.042) independently predicted major postoperative morbidities (Table 3(b)). Postoperative major morbidities were fivefold higher in patients with a preoperative platelet count <150 x 10^9^/L, but this difference was not significant (OR 4.9, 95% CI 0.9–26.3,* p*=0.062). Furthermore, multivariate analysis showed that postoperative 30-day mortality was fourfold higher in patients with thrombocytopenia compared with normal platelet counts (Table 3(c)) (OR 4.4, 95% CI 1.1–18.8,* p*=0.043).

After excluding patients with underlying cirrhosis (n = 7), we repeated univariate and multivariate analysis of PHLF, major morbidity and 30-day mortality. As presented in Supplementary Table S1, multivariate analysis demonstrated that PHLF (OR 5.7, 95% CI 2.6–12.8,* p*<0.001) and 30-day mortality (OR 5.9, 95% CI 1.3–27.1,* p*=0.021) were sixfold higher in patients with thrombocytopenia compared with those with normal platelet counts.

### 3.2. Patient Survival

**Figure 1:**
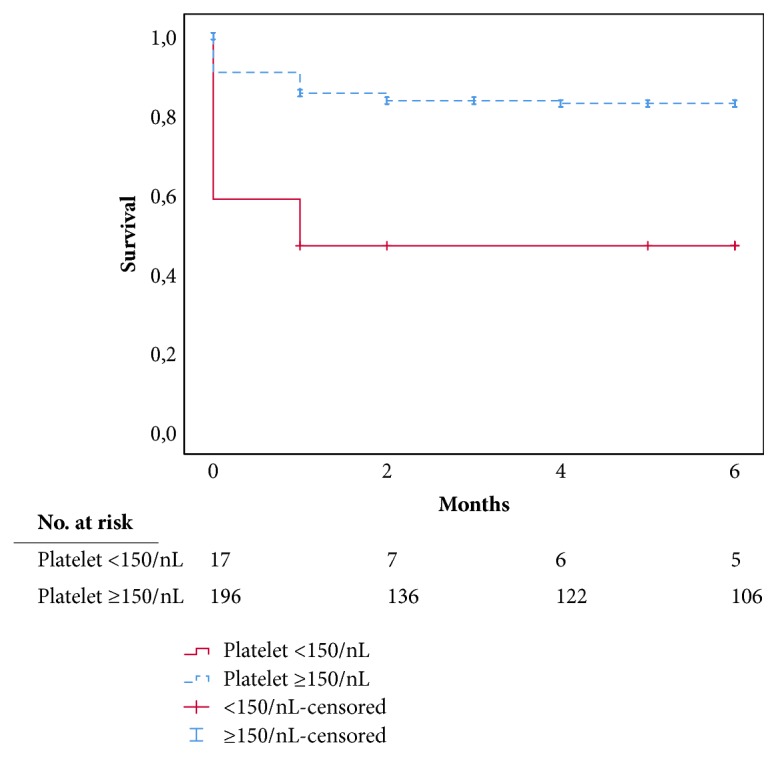
Six-month patient survival plot: significantly lower survival rates in patients with low preoperative platelet counts (<150 x 10^9^/L) compared with normal preoperative platelet counts (≥150) (log-rank test p<0.001).

The six-month survival rate was 80.1%±2.8% in our cohort. Patients with a preoperative platelet count <150 x 10^9^/L had a significantly lower six-month survival rate than patients with a preoperative platelet count ≥150 x 10^9^/L (Figure 1, 47.1%±12.1% versus 83.0%±2.8%, log-rank* p*<0.001).

## 4. Discussion

PHLF is a severe and potentially lethal complication after liver resection and is responsible for more than 60% of mortalities after EH [21, 22]. The high rate of mortality and morbidity following EH is a major concern in field of hepatobiliary surgery. Although low platelet count is associated with increased blood loss and longer operation time, it has been shown that low platelet counts can independently diminish postoperative liver regeneration and increase the risk of PHLF as well mortality [13, 23]. Recently, the effect of perioperative (preoperative or immediate postoperative) platelet counts on posthepatectomy morbidity and mortality has been investigated [13, 16, 24–28]. However, these studies investigated the predictive role of platelet count in all types of liver resection (minor, major, and extended) and did not distinguish between the different types of resection. PHLF and mortality rates are higher after EH, therefore we believe that the post-EH outcome is more clinically important and that the predictive role of platelet counts should be investigated separately following EH. To do this, we assessed the association of platelet counts and postoperative outcomes in a homogeneous subgroup of liver resection patients who underwent EH.

We demonstrated that a low preoperative platelet count is a predictive factor of PHLF and higher mortality after EH. In our series of EHs, the odds of development of PHLF and 30-day mortality in patients with low platelet counts were more than 4 and 6 fold higher than patients with normal platelet count, respectively. Moreover, our results showed that long-term survival was lower in patients with low platelet count than patients with normal platelet count. These findings indicate that a low platelet count independently predicts short- and long-term outcomes after EH. We selected a cut-off value of 150 x 10^9^/L for platelet counts because this is the minimum normal platelet count in our center and in most clinical settings.

In agreement with our findings, Alkozai et al. [9] reported a fourfold higher 90-day mortality rate after liver resection in colorectal metastasis patients with thrombocytopenia. They also reported delayed postoperative recovery of liver function in patients with low platelet count. However, in their study only 40% of patients underwent liver resection with remnant liver volume of <35%. Others have also shown that perioperative thrombocytopenia affects PHLF, morbidity, and mortality rates [24, 28]. Similar to our study, Venkat et al. [15], Maithel et al. [25], and Margonis et al. [26] evaluated the effect of perioperative platelet counts on PHLF and/or mortality with a cut-off value of 150 x 10^9^/L. Although these studies were not consistent in the platelet cut-off levels and type of liver resection they investigated, each has shown that platelet count (100 x 10^9^/L or 150 x 10^9^/L) is associated with posthepatectomy outcomes. In contrast, some authors have reported no significant association between perioperative platelet count and posthepatectomy outcomes [23, 29]. However, in these studies the platelet count was used as a continuous variable in univariate and/or multivariable analyses. In this regard, it is important to know that the exponentiated coefficient of a continuous predictor in logistic regression is the OR of a one-unit increase in the predictor. A one-unit change in platelet count is not clinically meaningful, which may explain why these authors did not find platelet counts to have significant predictive value.

The predictive role of platelets on PHLF and postoperative mortality may be explained by various mechanisms. One proposed mechanism is the direct promotion of liver regeneration by platelets [30]. This was first suggested by Tomikawa et al. [31] in 1996, who showed that platelets promote liver regeneration after resection by upregulating hepatocyte growth factor. Recent studies have shown that platelets secrete several bioactive factors including serotonin, vascular endothelial growth factor, platelet-derived growth factor, and tumor necrosis factors (TNF-*α* and TNF-*β*) to promote liver regeneration in a “direct way” [23, 32, 33]. Another possible mechanism is the “indicative role” of platelets for liver regeneration, which has been described by the parallel regulation of platelet production and liver regeneration by similar factors. Thrombopoietin and interleukin 6 regulate megakaryocyte maturation and platelet production, and can predict the postoperative patient outcome and trigger liver regeneration after hepatectomy [29, 34]. The risk of intra- and postoperative bleeding is increased in patients with thrombocytopenia. This increased blood loss can lead to additional hypoxic liver damage and therefore impaired hepatocyte function/regeneration. This could also explain PHLF and mortality in patients with thrombocytopenia. Surprisingly, Tomimaru and colleagues [14] showed that the platelet count is a better predictor of PHLF in hepatocellular carcinoma patients than the indocyanine green clearance test.

Furthermore, the association between low preoperative platelet count and high portal vein pressure suggests an alternative mechanism [25]. Low platelet counts in patients with liver disease may be secondary to the increased portal vein pressure and subsequent hypersplenism and increased thrombocyte sequestration in the spleen. Indeed, platelet counts can predict PHLF as an accurate and precise surrogate of the portal vein pressure. However, in our study none of the patients in the thrombocytopenia group had splenomegaly, portal hypertension, or cirrhosis. In addition, we performed a subgroup analysis in noncirrhotic patients to reveal the direct association of platelet count with PHLF and mortality.

The retrospective design is a limitation of the present study. However, platelet counts, other laboratory measurements, morbidity, and mortality of all consecutive patients were all recorded prospectively during the study period. To minimize potential bias and estimate the independent effect of the platelet count as accurately as possible, we controlled factors that are known to affect post-EH morbidity and mortality. These potentially confounding factors included age, gender, BMI, American Society of Anesthesiologists (ASA) score, indication of EH, intraoperative blood loss and transfusion, and operation time using univariate and multivariate regression analyses. Therefore, we evaluated the predictive role of platelet count independent of these factors. Cirrhosis is also associated with low platelet count so may confound the effect of thrombocytopenia on posthepatectomy outcome [35]. Therefore, we performed a subgroup analysis after exclusion of patients with cirrhosis.

## 5. Conclusions

In conclusion, preoperative thrombocytopenia seems to be a reliable predictor of PHLF and increased mortality after EH. This predictive role is independent of other related parameters, including age, cause of hepatectomy, intraoperative blood loss, and duration of surgery. Further randomized studies are required to evaluate the impact of increasing the preoperative platelet count (exogenous platelet infusion versus treatment of the underlying disease) on improving the postoperative outcomes after EH in patients with thrombocytopenia.

## Figures and Tables

**Table 1:** Clinicopathologic characteristics of patients who underwent extended hepatectomy.

**Variables**	**Total**
	**(n = 213)**
Age (years)	60.8 ±11.7
Gender	
Female/male	108/105
BMI (kg/m^2^)	25.53 ± 4.44
ASA score	
Class 1	4 (2.5%)
Class 2	76 (46.9%)
Class 3	82 (50.6%)

Cirrhosis	
Yes	7 (3.2%)
Indication of extended hepatectomy	
Benign liver disease	9 (4.2%)
Primary malignancy	123 (57.7%)
Cholangiocarcinoma	105 (85.4)
Hepatocellular carcinoma	18 (14.6%)
Metastatic disease	81 (38.0%)
Preoperative chemotherapy	
Yes	73 (35.8%)
Preoperative platelet count (x 10^9^/L)	
Mean (SD)	300.1 ± 121.5

Intraoperative blood loss (ml)	1638.21 ± 1535.49
Transfusion of RBC	
Patient	60 (31.1%)
Unit	1.52 ± 3.34
Transfusion of FFP	
Patient	44 (22.8%)
Unit	1.43 ± 3.64
Operation time (min)	293.78 ± 115.15

PHLF ^a^	56 (26.3%)
Grade A	16 (28.6%)
Grade B	14 (25.0%)
Grade C	26 (46.4%)
Major morbidity ^b^	57 (26.8%)
ICU stay (days)	8.14 ± 13.47
Hospitalization (days)	23.43 ± 16.68
30-day mortality	24 (11.3%)

BMI: body mass index; ASA: American Society of Anesthesiologists; SD: standard deviation; RBC: red blood cells; FFP: fresh-frozen plasma; PHLF: posthepatectomy liver failure; ICU: intensive care unit.

^a^ Based on the ISGLS definition.

^b^ Grades III and IV based on the Clavien-Dindo classification.

**Table 2:** Clinicopathologic characteristics of patients with a preoperative platelet count of <150 x 10^9^/L.

**Variables**	**Total**
	**(n = 17)**
Age (years)	63.1 ±12.5
Gender	
Female/male	8/9
BMI (kg/m^2^)	25.08 ± 3.88
ASA score	
Class 1	0 (0.0%)
Class 2	8 (57.1%)
Class 3	6 (42.9%)

Cirrhosis	
Yes	0 (0.0%)
Indication of extended hepatectomy	
Benign liver disease	2 (11.8%)
Primary malignancy	9 (52.9%)
Cholangiocarcinoma	9 (100%)
Hepatocellular carcinoma	0 (0.0%)
Metastatic disease	6 (35.3%)
Preoperative chemotherapy	
Yes	5 (29.4%)
Preoperative platelet count (x 10^9^/L)	
Mean (SD)	122.3 ± 22.3

Intraoperative blood loss (ml)	3352.94 ± 2019.32
Transfusion of RBC	
Patient	8 (50.0%)
Unit	4.38 ± 6.26
Transfusion of FFP	
Patient	9 (56.3%)
Unit	4.31 ± 5.91
Operation time (min)	381.29 ± 136.05

PHLF ^a^	10 (58.8%)
Grade A	0 (0.0%)
Grade B	2 (20.0%)
Grade C	8 (80.0%)
Major morbidity ^b^	6 (35.3%)
ICU stay (days)	16.65 ± 9.50
Hospitalization (days)	30.18 ± 15.20
30-day mortality	6 (35.3%)

BMI: body mass index; ASA: American Society of Anesthesiologists; SD: standard deviation; RBC: red blood cells; FFP: fresh-frozen plasma; PHLF: posthepatectomy liver failure; ICU: intensive care unit.

^a^ Based on the ISGLS definition.

^b^ Grades III and IV based on the Clavien-Dindo classification.

**Table:** (a) PHLF

**Variables**	**Univariate**			**Multivariate**		
	OR	95% CI	*p*	OR	95% CI	*p*
Age	1.015	0.988–1.043	0.280			
Gender	0.963	0.523–1.772	0.902			
BMI (kg/m^2^)	0.989	0.914–1.071	0.793			
ASA score	1.429	0.743–2.751	0.285			

Indication of extended hepatectomy						
Benign liver disease	Reference	Reference	Reference	Reference	Reference	Reference
Primary malignancy	1.367	0.256–7.290	0.714	0.810	0.120–5.470	0.828
Metastatic disease	2.306	1.159–4.591	0.017	1.897	0.806–4.463	0.141
Preoperative chemotherapy	1.679	0.723–3.896	0.228			
Platelet count <150 x 10^9^/L	4.658	1.678–12.929	0.003	4.351	1.266–14.953	0.020

Intraoperative blood loss (L)	1.159	1.036–1.297	0.010	0.884	0.615–1.271	0.506
Intraoperative RBC/FFP transfusion	2.617	1.343–5.098	0.005	2.226	0.859–5.769	0.099
Operation time (hour)	1.264	1.076–1.484	0.004	1.167	0.961–1.417	0.120

**Table:** (b) Major morbidity

**Variables**	**Univariate**			**Multivariate**		
	OR	95% CI	*p*	OR	95% CI	*p*
Age	1.036	1.010–1.062	0.006	1.050	1.020–1.083	0.001
Gender	1.148	0.669–1.969	0.617			
BMI (kg/m^2^)	1.011	0.944–1.083	0.758			
ASA score	1.329	0.752–2.346	0.328			

Indication of extended hepatectomy						
Benign liver disease	Reference	Reference	Reference	Reference	Reference	Reference
Primary malignancy	2.017	0.497–8.187	0.326	1.347	0.195–9.291	0.762
Metastatic disease	3.331	1.827–6.072	<0.001	2.387	1.111–5.127	0.026
Preoperative chemotherapy	0.626	0.274–1.428	0.266			
Platelet count <150 x 10^9^/L	10.427	2.321–46.845	0.002	4.923	0.922–26.296	0.062

Intraoperative blood loss (L)	1.365	1.181–1.577	<0.001	1.309	0.863–1.985	0.206
Intraoperative RBC/FFP transfusion	2.880	1.555–5.336	0.001	1.285	0.491–3.363	0.610
Operation time (hour)	1.431	1.215–1.685	<0.001	1.237	1.007–1.520	0.042

**Table:** (c) 30-day mortality

**Variables**	**Univariate**			**Multivariate**		
	OR	95% CI	*p*	OR	95% CI	*p*
Age	1.038	0.997–1.082	0.073	1.038	0.994–1.085	0.094
Gender	1.720	0.718–4.124	0.224			
BMI (kg/m^2^)	1.032	0.928–1.147	0.564			
ASA score	1.179	0.504–2.762	0.704			

Indication of extended hepatectomy						
Benign liver disease	Reference	Reference	Reference	Reference	Reference	Reference
Primary malignancy	2.406	0.239–24.220	0.456	1.784	0.078–40.796	0.717
Metastatic disease	3.517	1.150–10.754	0.027	3.460	0.856–13.980	0.081
Preoperative chemotherapy	0.570	0.126–2.568	0.464			
Platelet count <150 x 10^9^/L	5.394	1.784–16.311	0.003	4.430	1.055–18.777	0.043

Intraoperative blood loss (L)	1.342	1.085–1.659	0.007	1.166	0.753–1.805	0.492
Intraoperative RBC/FFP transfusion	3.216	1.295–7.986	0.012	1.604	0.435–5.921	0.478
Operation time (hour)	1.261	1.023–1.555	0.030	1.093	0.839–1.425	0.509

OR: odds ratio; CI: confidence interval; BMI: body mass index; ASA: American Society of Anesthesiologists; RBC: red blood cells; FFP: fresh-frozen plasma.

## Data Availability

The data used to support the findings of this study are available from the corresponding author upon request.

## Supplementary Materials

Supplementary Materials Supplementary Table S1. Subgroup analysis of predictive factors of PHLF, major morbidity, and 30-day mortality after extended hepatectomy in patients without cirrhosis.
