# Supplementary material for: Preoperative Thrombocytopenia May Predict Poor Surgical Outcome after Extended Hepatectomy
Source: Can J Gastroenterol Hepatol. 2018 Nov 1;2018:1275720. doi: 10.1155/2018/1275720 (PMC6236772; doi:10.1155/2018/1275720)
Supplement: Supplementary Materials — Supplementary Table S1. Subgroup analysis of predictive factors of PHLF, major morbidity, and 30-day mortality after extended hepatectomy in patients without cirrhosis. [file 1275720.f1.docx]

| **Supplementary Table S1. Subgroup analysis of predictive factors of PHLF, major morbidity, and 30-day mortality after extended hepatectomy in patients without cirrhosis**   1. **PHLF** | | | | | | | |
| --- | --- | --- | --- | --- | --- | --- | --- |
| **Variables** | **Univariate** | | |  | **Multivariate** | | |
|  | OR | 95% CI | *p* |  | OR | 95% CI | *p* |
| Age | 1.013 | 0.988–1.041 | 0.355 |  |  |  |  |
| Gender | 0.905 | 0.484–1.691 | 0.753 |  |  |  |  |
| BMI (kg/m^2^) | 0.993 | 0.915–1.079 | 0.871 |  |  |  |  |
| ASA score | 1.414 | 0.722–2.769 | 0.313 |  |  |  |  |
| Indication of extended hepatectomy   - Benign liver disease - Primary malignancy - Metastatic disease | Reference  1.367  2.241 | Reference  0.256–7.290  1.118–4.495 | Reference  0.714  0.023 |  | Reference  0.647  1.607 | Reference  0.092–4.569  0.659–3.920 | Reference  0.663  0.297 |
| Preoperative chemotherapy | 1.333 | 0.543–3.275 | 0.530 |  |  |  |  |
| Platelet count <150 x 10^9^/L | 4.850 | 1.742–13.505 | 0.003 |  | 5.739 | 2.574–12.796 | <0.001 |
| Intraoperative blood loss (L) | 1.395 | 1.104–1.762 | 0.005 |  | 0.841 | 0.574–1.234 | 0.376 |
| Intraoperative RBC/FFP transfusion | 2.717 | 1.384–5.335 | 0.004 |  | 2.201 | 0.803–6.030 | 0.125 |
| Operation time (hour) | 1.292 | 1.095–1.525 | 0.002 |  | 1.489 | 0.981–1.489 | 0.076 |
|  | | | | | | | |

| 1. **Major morbidity** | | | | | | | |
| --- | --- | --- | --- | --- | --- | --- | --- |
| **Variables** | **Univariate** | | |  | **Multivariate** | | |
|  | OR | 95% CI | *p* |  | OR | 95% CI | *p* |
| Age | 1.036 | 1.010–1.062 | 0.006 |  | 1.038 | 1.007–1.070 | 0.015 |
| Gender | 1.257 | 0.727–2.176 | 0.413 |  |  |  |  |
| BMI (kg/m^2^) | 1.025 | 0.955–1.100 | 0.498 |  |  |  |  |
| ASA score | 1.464 | 0.820–2.614 | 0.198 |  |  |  |  |
| Indication of extended hepatectomy   - Benign liver disease - Primary malignancy - Metastatic disease | Reference  2.017  3.702 | Reference  0.497–8.187  2.014–6.805 | Reference  0.326  <0.001 |  | Reference  1.019  2.460 | Reference  0.156–6.648  1.141–5.302 | Reference  0.984  0.022 |
| Preoperative chemotherapy | 0.683 | 0.294–1.586 | 0.375 |  |  |  |  |
| Platelet count <150 x 10^9^/L | 10.000 | 2.224–44.964 | 0.003 |  | 3.069 | 0.814–6.915 | 0.072 |
| Intraoperative blood loss (L) | 1.867 | 1.392–2.505 | <0.001 |  | 1.251 | 0.815–1.919 | 0.305 |
| Intraoperative RBC/FFP transfusion | 2.669 | 1.435–4.964 | 0.002 |  | 1.149 | 0.427–3.092 | 0.783 |
| Operation time (hour) | 1.431 | 1.212–1.689 | <0.001 |  | 1.243 | 1.008–1.531 | 0.041 |
|  | | | | | | | |

| 1. **30-day mortality** | | | | | | | |
| --- | --- | --- | --- | --- | --- | --- | --- |
| **Variables** | **Univariate** | | |  | **Multivariate** | | |
|  | OR | 95% CI | *p* |  | OR | 95% CI | *p* |
| Age | 1.037 | 0.995–1.080 | 0.088 |  | 1.037 | 0.992–1.084 | 0.109 |
| Gender | 1.716 | 0.708–4.164 | 0.232 |  |  |  |  |
| BMI (kg/m^2^) | 1.039 | 0.931–1.160 | 0.495 |  |  |  |  |
| ASA score | 1.345 | 0.557–3.264 | 0.510 |  |  |  |  |
| Indication of extended hepatectomy   - Benign liver disease - Primary malignancy - Metastatic disease | Reference  2.406  3.536 | Reference  0.239–24.220  1.149–10.877 | Reference  0.456  0.028 |  | Reference  2.121  3.541 | Reference  0.109–41.404  0.874–14.355 | Reference  0.620  0.077 |
| Preoperative chemotherapy | 0.631 | 0.139–2.864 | 0.551 |  |  |  |  |
| Platelet count <150 x 10^9^/L | 5.519 | 1.814–16.789 | 0.003 |  | 5.954 | 1.307–27.121 | 0.021 |
| Intraoperative blood loss (L) | 1.504 | 1.146–1.976 | 0.003 |  | 0.994 | 0.606–1.631 | 0.982 |
| Intraoperative RBC/FFP transfusion | 2.868 | 1.139–7.223 | 0.025 |  | 1.922 | 0.504–7.321 | 0.338 |
| Operation time (hour) | 1.244 | 1.006–1.539 | 0.044 |  | 1.091 | 0.836–1.423 | 0.522 |
| _OR: odds ratio; CI: confidence interval; BMI: body mass index; ASA: american society of anesthesiologists; RBC: red blood cells; FFP: fresh-frozen plasma_ | | | | | | | |
